# Robotically assisted spondylodesis for left-sided herniated disc L4–5 and spondylolysis L5–S1

**DOI:** 10.3171/2025.4.FOCVID2512

**Published:** 2025-07-01

**Authors:** Daniela Pierscianek, Ahmed Alfarra, Ralf Stroop, Tim W. Lau, Nicolai El Hindy

**Affiliations:** 1 Department of Neurosurgery and Spine Surgery and; 2 Corporate Communications, St. Marien Hospital Lünen, St. Paulus Gesellschaft, North Rhine-Westphalia, Germany

## Abstract

Robotic spine surgery is a rapidly developing field and offers several advantages for both patients and the surgical team. Several studies have shown greater accuracy in screw placement, less blood loss, less radiation exposure, and fewer reoperations compared with freehand procedures. Here the authors present the case of a 40-year-old woman with a herniated disc at level L4–5 and spondylolysis at level L5–S1. They performed robot-assisted spondylodesis using midline cortical screws in both levels due to foot-drop paralysis and refractory pain and present their workflow for robot-assisted spondylodesis.

The video can be found here: https://stream.cadmore.media/r10.3171/2025.4.FOCVID2512

## Transcript

### 0:20 Medical History.

We present the case of a patient with a herniated disc at L4–5 on the left and spondylolysis at L5–S1. The patient is 40 years old and has had repeated episodes of low back pain. During the last 3 weeks, she experienced left-sided lumboischialgia along the S1 nerve root and a severe foot drop grade 3 according to Medical Research Council (MRC) scale. Conservative treatment including oral pain medication and physical therapy carried out to date has not been successful.

### 0:57 MRI and CT.

The MRI shows the herniated disc in L4–5 on the left side with compression of the L5 root on the left and the listhesis L5–S1. No spinal canal stenosis is shown. The spondylolysis in L5–S1 can be easily visualized in the CT scan. The functional radiographs show no significant macroscopic mobility in the L5–S1 segment.

### 1:26 Patient Positioning.

The operation is carried out using the ExcelsiusGPS (Globus Medical). The patient is positioned in prone position, chest and pelvis are padded, and both legs are supplied with alternating pressure pumps. Special attention should be paid to the pressure-free positioning of the eyes. The surgical site is then washed sterilely and draped. The operating table is covered 360° and the x-ray is also draped sterilely.

### 1:56 DRB (Dynamic Reference Base) and Surveillance Marker.

The navigation star (DRB) and the surveillance marker are placed on the right and left side of the iliac crest. The camera is placed at the feet of the patient, and the surveillance marker monitors the position of the navigation star. Therefore, the stable position at the iliac crest is crucial for all following working steps.

### 2:24 X-Ray and CT Scan Matching.

X-ray images of the two segments are then taken in the AP (anteroposterior) and lateral beam path and transmitted to the surgical robot. To enable matching between CT scan and x-ray, each vertebral body is localized and defined in the AP and lateral x-ray views. Afterward, the robot’s software will perform a matching of the current x-rays and the preoperative CT images. Then, it delivers a 3D image for the navigation.

The surgeon verifies the matching individually for each segment, both in the AP and lateral beam path. The accuracy of the match is essential for the correct placement of the implants. In case of inaccuracy, the surgeon has to repeat the x-ray imaging. To plan the skin incision, the robotic arm displays the individual screw positions and the surgeon marks them on the skin. This enables small skin incisions to be made. In this case, the length of the skin opening is 6 cm. After the skin incision, the subcutaneous tissue is dissected and the muscle fascia is incised, the autochthonous muscles are pushed off and a double-barreled retractor is inserted.

### 3:52 Screw Placement.

The ExcelsiusGPS uses optical navigation. The screw trajectories and the trajectory of the cages are planned preoperatively using the preoperative CT dataset. The robot arm contains the end effector, which serves as active navigation, and, after verification, the instruments are used as navigated instruments that are inserted through the robot working channel. The navigation star is always oriented toward the camera.

A pedal is used to move the robot arm to the planned trajectory for the selected screw. When using the navigated instruments, a frame is displayed on the navigation and a colored traffic light system indicates the deviation from the planned trajectory. You can also see the position of the instruments compared to the planned trajectory directly on the monitor.

Now, the screw placement begins. This is performed in 4 steps. Using a high-speed drill, a pilot drill hole is created to prevent skiving on the bone, followed by drilling the pedicle, and finally the tap is applied. After these steps, the screw is inserted. As the screws can be exactly planned, the S1 screws are safely planned and inserted bicortical, which is crucial for the stability.

In this sequence, you can see the use of the pilot drill, which drills through the facet joint, and after drilling the pedicle and tapping, the screw is placed—guided by the robot arm and visualized at the monitor.

Several studies showed that the positioning of the screw using a robotic system is more accurate than freehand or navigated positioning of a screw.^1–3^

### 6:02 Midline Cortical Screws.

We use midline cortical screws (MCSs), also known as cortical bone trajectory (CBT) screws. They have been shown in several studies to have a higher primary stability and a higher pullout strength than traditional pedicle screws.^4,5^ The smaller surgical access with less soft tissue damage is also an advantage of these screws. This is shown schematically in the two figures.

### 6:26 Microsurgical Decompression and Removal of Disc Herniation.

After screw placement, the robotic arm is removed from the operating field and the rest of the operation is purely navigated. Using a microscope, a microsurgical decompression with laminectomy and complete bilateral facetectomy in both segments is performed. After mobilization of the dural sac at the level of L4–5 on the left, the herniated disc is exposed, mobilized, and removed.

### 7:02 Cage (Transforaminal Lumbar Interbody Fusion) Placement.

After decompression, the disc space is incised and then cleared out using navigated shavers and curettes. After preparation of the endplates, the space is filled with bone and osteoinductive material. The navigated cage is then inserted. The cage holder is also a navigated instrument, and the position of the cage can be followed precisely during insertion. As an example, the cage L5–S1 is inserted from the right side and precisely aligned using the navigation. The navigated cage is expandable. As soon as the planned position is arrived, the cage can be individually expanded.

### 7:58 Rods, Reduction of Listhesis, and Compression.

After screw and cage placement, the screw heads are applied. During decompression and cage placement, the heads of the screws would hinder the work; therefore, they are applied afterward.

Then, the rod is fitted in. To achieve a reduction of the spondylolisthesis, the rod is inserted at S1 and fixed with locking screws, and then the reduction cuffs are inserted in L5. Once all the locking screws have been inserted, they are locked with a counterholder and torque wrench and are made monoaxial so that the two segments can be compressed.

### 8:56 Wound Closure.

A drain is inserted and the wound is then closed in layers. The skin is sutured intracutaneously. The wound is infiltrated with a local anesthetic. The patient is extubated in the operating theater and then transferred to the normal ward. The blood loss was < 250 mL.

### 9:17 Postoperative Outcome.

Before the patient is repositioned, AP and lateral x-ray are carried out. Immediately after the operation, the foot drop is already improved and the radiating pain and the tingling paresthesia are gone. The patient is mobilized on the same day.

In our view, robotic assisted surgery improves the accuracy of screw and cage placement, shortens the operating time, reduces blood loss, and minimizes x-ray exposure.

### Acknowledgments

We thank Heike Schulte for assisting in the operation.

## Disclosures

Dr. El Hindy reported consulting for Globus Medical, giving talks about the robotic platform, and his institution is a preceptor site for the robotic platform.

## Author Contributions

Primary surgeon: Pierscianek. Assistant surgeon: Alfarra, Lau. Editing and drafting the video and abstract: Pierscianek, Lau. Critically revising the work: all authors. Reviewed submitted version of the work: Pierscianek, Alfarra, Lau, El Hindy. Approved the final version of the work on behalf of all authors: Pierscianek. Supervision: Pierscianek, El Hindy.

## Supplemental Information

### Patient Informed Consent

The necessary patient informed consent was obtained in this study.
